# Prediction of Immune-Checkpoint Blockade Monotherapy Response in Patients With Melanoma Based on Easily Accessible Clinical Indicators 

**DOI:** 10.3389/fonc.2021.659754

**Published:** 2021-05-27

**Authors:** Hwa Kyung Byun, Jee Suk Chang, Minkyu Jung, Woong Sub Koom, Kee Yang Chung, Byung Ho Oh, Mi Ryung Roh, Kyung Hwan Kim, Choong-Kun Lee, Sang Joon Shin

**Affiliations:** ^1^ Department of Radiation Oncology, Yonsei Cancer Center, Yonsei University College of Medicine, Seoul, South Korea; ^2^ Division of Medical Oncology, Department of Internal Medicine, Yonsei University College of Medicine, Seoul, South Korea; ^3^ Department of Dermatology, Yonsei University College of Medicine, Seoul, South Korea

**Keywords:** lymphopenia, overall survival, predictor, melanoma, immune checkpoint blockade

## Abstract

**Background:**

Immune checkpoint blocker (ICB) has shown significant clinical activity in melanoma. However, there are no clinically approved biomarkers to aid patient selection. We aimed to identify patients with advanced or metastatic melanoma who are likely to benefit from ICB monotherapy using easily accessible clinical indicators.

**Materials and Methods:**

We retrospectively reviewed the records of 134 patients with advanced or metastatic melanoma who received ICB monotherapy between 2014 and 2018. Prognostic factors of overall survival (OS) and progression-free survival (PFS) were determined using Cox regression analysis.

**Results:**

During the median follow-up of 13.7 months, the median OS and PFS were 18.4 and 3.4 months, respectively. Visceral/central nervous system (CNS) metastasis (OS: adjusted hazards ratio [HR], 1.82; p=.014; PFS: HR, 1.59; p=.024), lymphopenia (<1000 cells/µL) within 3 months (OS: HR, 1.89, p=.006; PFS: HR, 1.70; p=.010), and elevated baseline lactate dehydrogenase (LDH) level (OS: HR, 2.61; p<.001; PFS: HR, 2.66; p<.001) were independent prognostic factors for both poor OS and PFS. Development of immune-related adverse events (irAE; e.g., hypothyroidism or vitiligo) within 6 months showed a trend toward better OS in multivariable analysis (HR, 0.37; p=.058). Patients with normal LDH levels and no visceral/CNS metastasis had a substantially better OS than the others (median, 40.4 *vs.* 13.6 months; p<.001). Among others, patients who developed irAE within 6 months achieved long-term OS (median, 43.6 *vs.* 13.1 months; p=.008). A decision tree was suggested using four risk factors, and the risk stratification provided significant distinction between the survival curves.

**Conclusion:**

The four easily accessible clinical indicators associated with better treatment outcomes after ICB monotherapy in patients with advanced or metastatic melanoma were LDH level, the extent of disease, lymphopenia, and irAE. The combined use of these indicators can be clinically useful in improving risk stratification of patients treated with ICB monotherapy.

## Introduction

The recent emergence of cancer immunotherapies has led to a significant shift in the clinical management of metastatic melanoma (1). Prior to 2011, patients with advanced or metastatic melanoma only had palliative treatment solutions that offered little to no survival benefit. Patients now benefit from novel immune checkpoint blockers (ICBs)—anti-cytotoxic T-lymphocyte protein 4 (CTLA-4) and anti-programmed cell death protein 1 (PD-1). In recent years, several randomized controlled phase III trials have shown the efficacy and safety of ICBs. The CheckMate 066 trial showed that nivolumab was associated with significant improvements in overall survival (OS) and progression-free survival (PFS) compared with dacarbazine in treatment-naïve patients who had metastatic melanoma without a BRAF mutation (2). Patients with advanced melanoma who received ipilimumab plus dacarbazine had better OS than those who received dacarbazine alone (3). Patients with metastatic melanoma who received ipilimumab plus gp100 peptide vaccine had improved OS than those who received gp100 alone (4). Furthermore, the CheckMate 067 and KEYNOTE-006 trials showed that nivolumab and pembrolizumab, respectively, improved treatment outcomes more than ipilimumab (5, 6). The clinical effects of ICBs are most apparent in patients with metastatic or advanced melanoma (2–7). However, response rates are modest with ICB monotherapy. The response rates for ipilimumab and pembrolizumab or nivolumab range from 11% to 19% (5, 7) and 33% to 44% (2, 5, 6), respectively. We have previously reported that the objective response rate of ICB monotherapy in South Korean patients with melanoma is 15% (8).

Currently, there are no clinically approved biomarkers to aid patient selection for immunotherapy in melanoma. Recent studies have been focusing on identifying biomarkers that could predict patient response prior to treatment initiation or very early during the treatment course to maximize therapeutic efficacy, minimize medical costs, and overcome side effects  (9). In recent years, studies have been conducted on biomarkers using omics technologies such as next-generation sequencing and mass cytometry. With these novel markers, more easily accessible clinical indicators that can be identified by a simple blood test or routine clinical examination may ideally aid in therapeutic decision making. These indicators may be useful while integrating omics technologies in actual practice. Thus, we sought to identify patients with advanced or metastatic melanoma who are likely to benefit from ICB monotherapy using easily accessible clinical indicators.

## Materials and Methods

### Patient Population

We retrospectively identified consecutive patients with advanced or metastatic malignant melanoma treated with ICBs between 2014 and 2018 at Yonsei Cancer Center, Seoul, Republic of Korea. Data regarding patient demographics, treatments and related parameters, and outcomes were obtained from the medical records. The inclusion criteria were as follows: age ≥18 years; pathologically confirmed malignant melanoma; at least one cycle of treatment with a CTLA-4 inhibitor or a PD-1 inhibitor; and availability of follow-up images for the assessment of treatment response. The tumors were clinically classified according to the extent of surrounding solar elastosis and the anatomical site as chronic sun-damaged melanomas and non-chronic sun-damaged melanomas (acral, mucosal, and uveal). The use of palliative radiotherapy during treatment was evaluated for all patients. For advanced or metastatic malignant melanoma, palliative radiotherapy was administered for symptomatic metastasis after a thorough discussion with a multidisciplinary melanoma team, including medical oncologists, radiation oncologists, dermatologists, ophthalmologist, and pathologists. Combination therapy with an ICB and radiotherapy was defined as the administration of radiotherapy during ICB therapy or within 3 months before and after ICB therapy. This study was approved by our Institutional Review Board (4–2019–0796). The requirement for informed consent was waived for this retrospective study.

### Outcome Assessment

The follow-up period was defined as the interval between the first ICB administration and the date of the last visit or death. PFS was defined as the time from ICB administration to disease progression or death, and OS was defined as the time from ICB administration to death from any cause. The best radiological response was assessed based on the immune Response Evaluation Criteria in Solid Tumors.

Adverse events were recorded using the Common Terminology Criteria for Adverse Events, version 4.03. Because immunotherapy-related hypothyroidism or vitiligo is associated with favorable treatment outcomes in patients with melanoma (10, 11) and treatment-related lymphopenia is associated with poor treatment outcomes in various types of cancer including melanoma  (12–14), we included these adverse events in the subsequent analyses to identify prognostic factors. The extent of disease was classified using the M stage of cutaneous melanoma in the 8^th^ edition of AJCC, where M1a indicates metastasis to skin and soft tissue including muscle and/or nonregional lymph nodes, M1b indicates metastasis to the lungs, M1c indicates metastasis to non-central nervous system (CNS) visceral sites, and M1d indicates metastasis to the CNS. Patients were stratified into groups with visceral or CNS metastasis (M1c or d) and others.

### Statistical Analysis

Cox’s regression model was used for univariable and multivariable analyses of PFS and OS. Factors with a P-value of <0.1 in univariable analyses were included in multivariable analysis. Survival analyses were performed using the Kaplan–Meier method, and the log-rank test was used for the intergroup comparisons. A p-value of <0.05 was considered statistically significant. Statistical analyses were performed using SPSS (version 25; IBM Inc., Armonk, NY, USA).

## Results

### Patient, Tumor, and Treatment Characteristics

The patient, tumor, and treatment characteristics are summarized in **Table 1**. The median age at referral for ICB therapy was 60 years (range, 18–88 years). Of the 134 patients, 107 (79.9%), 22 (16.4%), and 5 (3.7%) were diagnosed with acral/mucosal, uveal, and chronic sun damage subtypes, respectively. The extent of disease was as follows: locally unresectable in 8 (6%) patients; skin, soft tissue, and/or regional nodal metastasis in 32 (24%) patients; lung metastasis in 18 (13.4%) patients; non-CNS visceral organ metastasis in 68 (50.7%) patients; and CNS metastasis in 8 (6%) patients. The median baseline lactate dehydrogenase (LDH) level was 215 U/L (range, 134–3002 U/L). The baseline LDH level was elevated in 45 (33.6%) patients. The median number of ICB cycles was 3 (range, 1–33). Pembrolizumab (n=96, 71.6%) was the most commonly used medication, followed by ipilimumab (n=31, 23.1%) and nivolumab (n=7, 5.2%). Combination therapy with an ICB and palliative radiotherapy was used in 63 (47.0%) patients.

**Table 1:** Patient, tumor, and treatment characteristics.

	N=134
Age at ICB administration, median (range)	59.8 (18.4–87.8)
Sex, n (%)	
Male	66 (49.3)
Female	68 (50.7)
Subtype, n (%)	
Acral/mucosal	107 (79.9)
Uveal	22 (16.4)
Chronic sun damage	5 (3.7)
BRAF mutation, n (%)	
Wild type	93 (69.4)
V600 mutation	16 (11.9)
Unknown	25 (18.7)
Disease extent, n (%)	
Locally unresectable status	8 (6)
Skin, soft tissue, regional nodal metastasis	32 (23.9)
Lung metastasis	18 (13.4)
Non-CNS visceral site metastasis	68 (50.7)
CNS metastasis	8 (6)
Baseline LDH, median (range), U/L	215 (134–3002)
Baseline LDH, n (%)	
normal	82 (61.2)
elevated	45 (33.6)
missing	7 (5.2)
No. of previous systemic therapies, n (%)	
0	87 (64.9)
1	24 (17.9)
2	20 (14.9)
≥3	3 (2.2)
Cycles of ICB, median (range)	3 (1–33)
ICB type, n (%)	
Ipilimumab	31 (23.1)
Pembrolizumab	96 (71.6)
Nivolumab	7 (5.2)
Radiotherapy, n (%)	
No	71 (53)
Yes	63 (47)

CNS, central nervous system; ICB, immune checkpoint blocker; LDH, lactate dehydrogenase.

### Immunotherapy-Related Adverse Events

During follow-up, grade 1/2 immunotherapy-related hypothyroidism and vitiligo developed in 9 (6.7%) and 15 (11.2%) patients, respectively, of which 6 (4.5%) and 8 (6.0%) patients manifested the adverse events within 6 months, respectively. Of the nine patients who developed immune-related hypothyroidism after ICB monotherapy, all patients were treated with levothyroxine and three were additionally treated with glucocorticoids. The OS and PFS were not significantly different between the patients who received glucocorticoids and those who did not (median OS, 22.4 months *vs.* not reached; p=.271; median PFS, 2.4 months *vs.* 2.8 months; p=.807). Of note, the published literature has shown that although the theoretical risk that immunosuppression reduces antitumor efficacy has not been proven, immunosuppression does carry additional risks that clinicians should consider  (15). Of the 15 patients who developed immune-related vitiligo, none of them was treated for vitiligo.

Other adverse events were grade 1/2 skin rash (n=7, 5.2%), grade 3/4 liver enzyme elevation (n=2, 1.5%), grade 3 thrombocytopenia (n=1, 0.7%), and grade 1 pruritus (n=1, 0.7%). Within 3 months, lymphopenia developed in 51 (38.1%) patients, of which 12 (9.0%), 19 (14.2%), 16 (11.9%), and 4 (3.0%) patients developed grade 1, 2, 3, and 4 lymphopenia, respectively.

### Identification of Prognostic Factors

We conducted univariable and multivariable analyses for OS and PFS using patient, tumor, and treatment characteristics and immunotherapy-related adverse events (irAE) to identify prognostic factors in patients with melanoma treated with ICB (**Table 2**). In univariable analyses for OS, visceral or CNS metastasis, high baseline LDH level, lymphopenia within 3 months, and hypothyroidism or vitiligo within 6 months were significantly associated with OS (all P<0.05). Multivariable analysis for OS revealed that visceral or CNS metastasis (hazards ratio [HR], 1.82; 95% CI, 1.09–3.04; p=0.014), high baseline LDH level (HR, 2.61; 95% CI, 1.59–4.28; p<0.001), and lymphopenia within 3 months (HR, 1.89; 95% CI, 1.16–3.09; p=0.006) were independent prognostic factors for OS. Hypothyroidism or vitiligo within 6 months had marginal significance in multivariable analysis (HR, 0.37; 95%, 0.13–1.04; p=0.058). The same factors were independent prognostic factors for PFS. Visceral organ or CNS metastasis (HR, 1.59; 95% CI, 1.06–2.39; p=.024), high baseline LDH level (HR, 2.66, 95% CI, 1.71–4.13; p<0.001), and lymphopenia within 3 months (HR, 1.70; 95% CI, 1.13–2.54; p=.010) were independently associated with poor PFS.

**Table 2:** Analysis of factors associated with progression-free survival and overall survival.

	Overall survival				Progression-free survival			
	Univariable analysis		Multivariable analysis		Univariable analysis		Multivariable analysis	
	HR (95% CI)	P	HR (95% CI)	P	HR (95% CI)	P	HR (95% CI)	P
Age (per 1 year increase)	1.01 (0.99–1.03)	0.563			0.99 (0.97–1.01)	0.328		
Female (*vs.* Male)	1.04 (0.67–1.62)	0.867			0.95 (0.66–1.37)	0.778		
Subtype								
Uveal (*vs.* acral/mucosal)	1.39 (0.79–2.45)	0.258			0.84 (0.51–1.39)	0.505		
Chronic sun damage (*vs.* acral/mucosal)	0.79 (0.19–3.23)	0.739			0.5 (0.16–1.61)	0.245		
BRAF mutation								
V600 mutation (*vs.* wild type)	0.66 (0.31–1.4)	0.282			1.66 (0.96–2.86)	0.068	1.13 (0.63–2.02)	0.692
Unknown (*vs.* wild type)	1.37 (0.77–2.42)	0.286			0.8 (0.49–1.33)	0.395	0.63 (0.36–1.09)	0.098
ICB type								
Pembrolizumab (*vs.* ipilimumab)	0.92 (0.54–1.57)	0.769			0.76 (0.49–1.17)	0.211		
Nivolumab (*vs.* ipilimumab)	0.72 (0.25–2.09)	0.547			0.46 (0.18–1.19)	0.108		
No. of previous chemotherapy (per 1 increase)	1.06 (0.85–1.32)	0.618			1.2 (0.97–1.47)	0.089	1.20 (0.97–1.50)	0.099
Radiotherapy yes (*vs.* no)	1.29 (0.83–2.01)	0.264			0.94 (0.65–1.36)	0.746		
Visceral organ/CNS metastasis yes (*vs.* no)	2.44 (1.5–3.97)	<0.001	1.82 (1.09–3.04)	0.014	1.72 (1.17–2.52)	0.006	1.59 (1.06–2.39)	0.024
Baseline LDH elevation yes (*vs.* no)	3.51 (2.2–5.59)	<0.001	2.61 (1.59–4.28)	<0.001	3.03 (2.01–4.58)	0.000	2.66 (1.71–4.13)	<0.001
Lymphopenia within 3 months yes (*vs.* no)	2.48 (1.57–3.93)	<0.001	1.89 (1.16–3.09)	0.006	1.86 (1.28–2.73)	0.001	1.70 (1.13–2.54)	0.010
Hypothyroidism or vitiligo within 6 months yes (*vs.* no)	0.35 (0.14–0.87)	0.023	0.37 (0.13–1.04)	0.058	0.62 (0.33-1.16)	0.135	0.72 (0.36–1.42)	0.338

CI, confidence interval; CNS, central nervous system; HR, hazards ratio; ICB, immune checkpoint blocker; LDH, lactate dehydrogenase.

Additionally, the identified prognostic factors were evaluated for their ability to predict the best radiological tumor response. Twenty-four (17.9%) patients had an objective response (complete or partial response). No visceral/CNS metastasis [odds ratio (OR), 6.92; 95% CI, 2.40-19.96; p<.001] and normal baseline LDH level (OR, 3.31; 95% CI, 1.05-10.38; p=.040) significantly predicted objective response, while no lymphopenia (OR, 1.28; 95% CI, 0.51-3.26; p=.599) and immune-related adverse events (OR, 1.29; 95% CI, 0.33-5.01; p=.717) did not predict objective response.

### Survival Analysis

**Figure 1:**
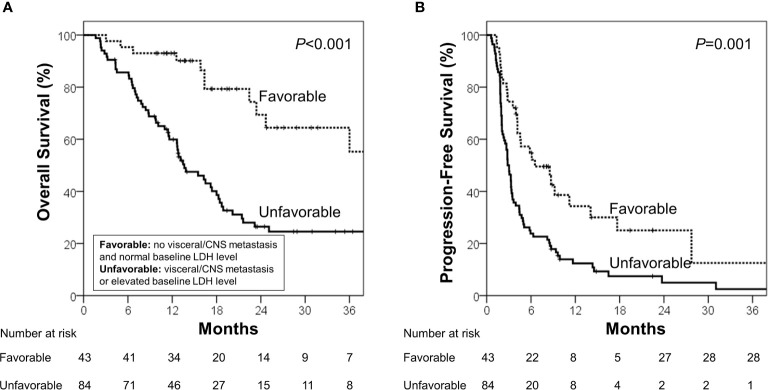
**(A)** Overall survival and **(B)** progression-free survival in the favorable and unfavorable groups. The favorable group was defined as patients with no visceral/CNS metastasis and normal baseline LDH level, and the unfavorable group was defined as other patients.

**Figure 2:**
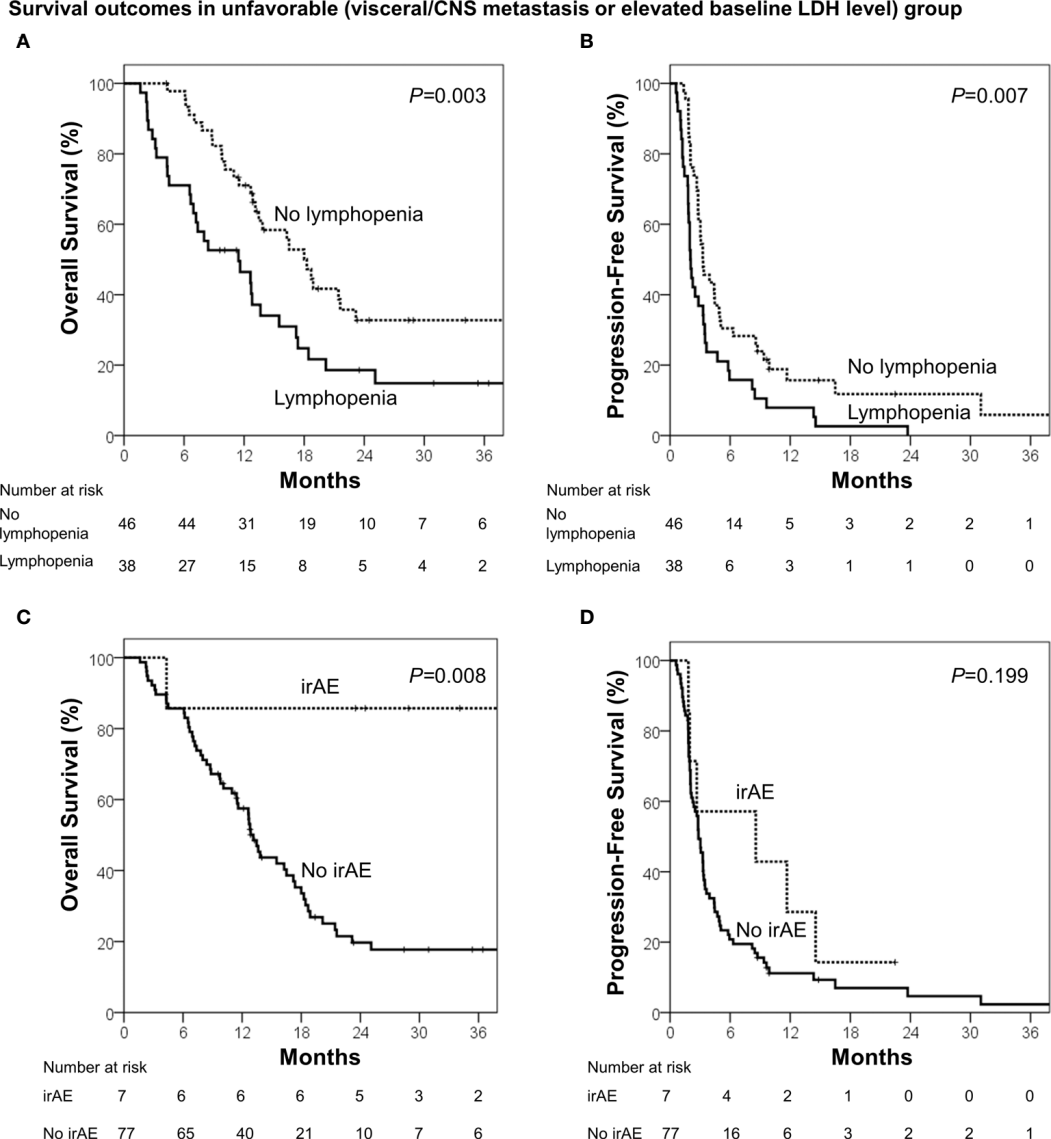
Survival analyses among the unfavorable group (visceral/CNS metastasis or elevated baseline LDH level). **(A)** Overall survival and **(B)** progression-free survival in patients who developed lymphopenia (<1000 cells/μL) within 3 months and those who did not. **(C)** Overall survival and **(D)** progression-free survival in patients who developed immune-related adverse events (irAE; hypothyroidism or vitiligo) within 6 months and who did not.

During the median follow-up of 13.7 months (range, 1.6–68.3 months), the median OS and PFS were 18.4 months [95% confidence interval (CI), 14.2–22.6 months] and 3.4 months (95% CI, 2.5–4.3 months), respectively. Using the identified risk factors, we divided patients into subgroups and conducted survival analyses. First, the extent of disease and baseline LDH levels were used to define the patient subgroup because these two factors can be identified prior to ICB administration. Patients with no visceral/CNS metastasis and normal baseline LDH levels were categorized into the favorable group (n=43) and the others were categorized into the unfavorable group (n=84). The favorable group had significantly better OS (median, 40.4 *vs.* 13.6 months; p<0.001) and PFS than the unfavorable group (median, 6.5 *vs.* 2.8 months; p=0.001; **Figure 1**). Because OS and PFS were relatively poor in the unfavorable group, it was important to further identify the subgroup of patients who may benefit from ICB therapy. Thus, we conducted the subsequent survival analyses in the unfavorable group (n=84). In the unfavorable group, the patients who did not develop lymphopenia within 3 months (n=46) had a significantly better OS (median, 18.3 *vs.* 11.4 months; p=.003) and PFS than those who developed lymphopenia (median, 3.3 months *vs.* 2.0 months; p=.007; **Figures 2A, B**). In the unfavorable group, a few patients who developed immune-related hypothyroidism or vitiligo within 6 months (n=7) had exceptionally better OS than those without such immune-related events (median, 43.6 *vs.* 13.1 months; p=.008), while PFS was not significantly different (median, 8.6 *vs.* 2.8 months; p=.199, **Figures 2C, D**).

Among all patients, those who developed hypothyroidism (n=6) and vitiligo (n=8) within 6 months had a median OS of 22.4 months and 43.6 months (p=.081) and a median PFS of 2.8 months and 8.6 months, respectively (p=.717).

### Clinical Decision Tree Proposal

**Figure 3:**
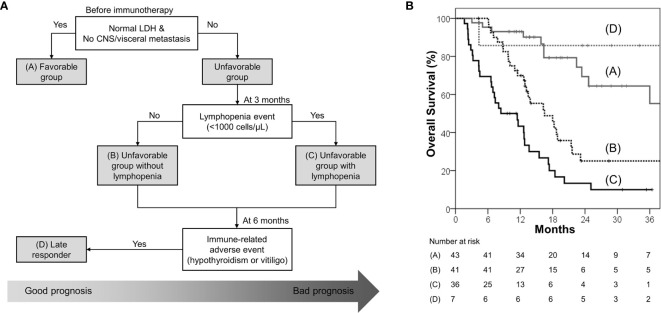
**(A)** Proposed decision tree to predict patients who may benefit from immune checkpoint blockade; it was used to determine the four risk groups: favorable group (patients with normal baseline LDH level and no CNS/visceral metastasis; group A), unfavorable group (patients with elevated baseline LDH level or CNS/visceral metastasis) without lymphopenia within 3 months (group B), unfavorable group with lymphopenia within 3 months (group C), and late responders (unfavorable group with immune-related hypothyroidism or vitiligo within 6 months; group D) **(B)** Overall survival according to the four risk groups.

A clinical decision tree proposal based on the survival analysis is shown in **Figure 3A**. Patients with normal baseline LDH levels and no visceral/CNS metastasis (favorable group) had exceptionally better OS and PFS than the others; hence, these patients are most likely to benefit from ICB therapy. Among the unfavorable group, patients without lymphopenia within 3 months had better OS and PFS; hence, these patients are more likely to benefit from ICB therapy than those with lymphopenia. A small group of patients who developed immune-related hypothyroidism or vitiligo within 6 months had exceptionally favorable OS; hence, these patients were classified as “late responders.” The risk stratification provided significant distinction between the Kaplan–Meier curves (**Figure 3B**).

Of the late responders, except 1 patient whose OS was 4.3 months, the other 6 patients had long-term OS, which ranged from 23.5 to 68.3 months. The PFS of these patients ranged from 2.0 to 34.1 months. Four of them underwent heavy subsequent treatments such as systemic chemotherapy and immunotherapy as well as local therapy such as gamma knife surgery, radiotherapy, and surgical resection. The profiles of the late responders are shown in **Supplementary Table 1**.

## Discussion

In this study, we identified four clinical indicators to identify patients with advanced or metastatic melanoma who may benefit from ICB monotherapy. These include baseline LDH level, the extent of disease, lymphopenia, and irAE. Among the favorable group who had normal baseline LDH level and no visceral/CNS metastasis, we observed very prolonged OS (median, 40.4 months) even if all patients had advanced or metastatic melanoma. Although patients were classified into the unfavorable group, subgroups of patients who may have longer OS can be sorted early during the treatment course using other clinical indicators, such as lymphopenia and irAE. Baseline LDH level, the extent of disease, and lymphopenia were independent prognostic factors for both OS and PFS.

As response to immunotherapy can be achieved for only a subset of patients, there is a crucial need to identify biomarkers to predict the efficacy of anti-CTLA-4 or anti-PD-1 treatment or identify a specific subset of patients who may benefit from immunotherapy. Researchers have investigated many potential biomarkers for immunotherapy in advanced or metastatic melanoma patients using novel technologies, such as next-generation sequencing, T-cell receptor profiling, and mass cytometry (9). Among them, tumor mutations and neoantigen load as well as the expression of immune-related genes in tumor tissue and/or the presence of CD8+ T-cell infiltrates showed significant correlations to response in large genetic and transcriptional analyses of tumor tissue (16). The response prediction would be more powerful if these high-technology novel biomarkers are combined with clinical indicators which can be obtained using non-invasive and easily accessible techniques. The suggested clinical biomarkers in the current study can be determined through a simple blood test or routine clinical exam and are cost-effective and quickly identified. Thus, we surmise that utilizing these easily accessible biomarkers will be of clinical use to guide proper patient selection for immunotherapy.

In 2009, LDH was shown to be an independent predictor of survival in melanoma and was therefore added to the AJCC guidelines (17). Accelerated metabolism in cancer cells requires increased glycolysis that produces elevated levels of LDH as a byproduct, which is therefore a robust proxy to assess tumor burden (18). In the context of immunotherapy, baseline LDH level and visceral/CNS metastasis were both independent prognostic factors in this study. This finding is consistent with previous reports. A retrospective data from the Netherlands and the United Kingdom also suggested that patients with metastatic melanoma whose baseline serum LDH was greater than twice the upper limit were unlikely to benefit from ipilimumab treatment (19). A retrospective large cohort of advanced melanoma patients also showed that elevated LDH and the presence of liver metastasis predict poor response to anti-PD-1 therapy (20).

We also revealed that patients who developed lymphopenia within 3 months after ICB initiation are associated with poor OS. Lymphocytes are important mediators of ICB mechanism. Given that circulating lymphocytes are the cells that eventually infiltrate tumors, their depletion might contribute to suboptimal treatment outcomes after immunotherapy. Similar to our results, a retrospective analysis with melanoma patients treated with ipilimumab also showed that increases in absolute lymphocyte counts at 2–8 weeks and circulating CD4+ and CD8+ T cells at 8–14 weeks were associated with positive clinical outcomes (21).

In this study, 7% and 11% of patients developed immunotherapy-related hypothyroidism and vitiligo, respectively, of which 5% and 6% developed within 6 months, respectively. Although irAE can present at any time, including after cessation of ICB therapy, we confined the biomarker to the irAE occurring within 6 months. Because it is difficult to make a clinical decision based on delayed irAE, we suppose that irAE occurring at least within 6 months would have clinical significance as a biomarker. A subgroup of patients who developed immune-related hypothyroidism or vitiligo in the unfavorable group had longer OS than other subgroups of patients (median, 43.6 *vs.* 13.1 months; p=.008), although PFS was not significantly longer (median, 8.6 *vs.* 2.8 months; p=.199). The findings are supported by previous studies. A systematic review showed that patients who developed vitiligo were associated with two to four times less risk of disease progression or death, respectively, compared to those without  (11). A prospective observational study of 67 melanoma patients showed that an objective response to treatment was associated with a higher incidence of vitiligo and all 17 patients who developed vitiligo were alive at the time of analysis, which means that patients with vitiligo had durable response. A retrospective study with 174 patients who received ICB for metastatic or advanced cancers showed a significantly longer PFS (median, 66 *vs.* 27 weeks) and OS (median, 156 *vs.* 59 weeks) in the thyroid dysfunction group than in the euthyroid group  (10). As this group had an extremely long survival even after disease progression, we named the group “late responders.” The subsequent treatments after ICB monotherapy had a long-term response (**Supplementary Table 1**). We did not elucidate whether the long-term OS was attributed to the irAE itself or the good treatment response. If the former is correct, irAE would be a prognostic factor, and if the latter is correct, irAE would be a predictive factor. Further study regarding this issue is warranted.

Using a simple decision tree, we suggested an optimal way to apply these factors in the clinical decision making in a time dependent manner. Nosrati et al. (20) developed a clinical scoring system to predict response to anti-PD-1 monotherapy in patients with advanced melanoma. The variables used in the scoring system were baseline clinical factors, such as sex, age, previous ipilimumab treatment, elevated LDH, and liver metastasis. Our model is practical in that it is not only simple but also includes variables of multiple time points, such as baseline, 3 months, and 6 months. This model has strength in that it can be applied before ICB monotherapy and during early ICB monotherapy.

Our study is retrospective in nature and therefore is limited by the presence of uncontrolled confounding factors, variations in ICB therapy cycles, variable radiation doses/sites, and under-reporting of toxicity. In addition, information regarding date of progression on ICB was not uniformly assessed because it would have been on a prospective clinical trial. Thus, we focused mainly on OS given the atypical patterns of response that can be observed after ICB therapy. Moreover, the sample size was relatively small. However, we collected a homogenous cohort of patients with advanced melanoma who received ICB therapy at a single institution. As the sample size was small, the correlation between the biomarkers and survival outcomes did not imply causation. Therefore, our findings need to be further validated using a larger global data set. Furthermore, irAE was significant in the univariable analysis for OS, but it lost its significance in the subsequent multivariable analysis, which may be due to the small number of groups. Additionally, these patients had stage IV or unresectable disease, so they did not undergo surgery or biopsy at the time of immunotherapy. Therefore, there was no tumor sample available at the time of immunotherapy to show molecular data or pathological images. We are currently conducting a prospective phase II trial on the efficacy of a combination of immunotherapy and radiotherapy for treating melanoma (NCT 04017897). In this trial, we are collecting blood samples, tumor tissues, and stool samples from participants and planning to analyze them.

In conclusion, we suggested a clinical predictive model using easily accessible clinical indicators to predict treatment outcomes in patients with advanced or metastatic melanoma who received ICB monotherapy. These indicators are baseline LDH level, visceral/CNS metastasis, lymphopenia within 3 months, and hypothyroidism or vitiligo within 6 months. Although these indicators have been reported as prognostic factors separately in previous reports, we showed that all were independent prognostic factors in a patient cohort, which add to the growing body of literature. The identification of a clinical predictive model is critical due to the following reasons. Firstly, it allows patients who are unlikely to benefit from anti-PD-1 therapy to be spared from unnecessary risk of toxicity and to rationally select a combination that will better fit them. Secondly, it can spare those who are likely to respond to PD-1 monotherapy from unnecessary toxicities from combination immunotherapy approach. This model could potentially have a role in the therapeutic decision-making and proper patient selection regarding immunotherapy. Its validation in future studies is warranted.

## Data Availability Statement

The datasets generated for this study are available on request to the corresponding author.

## Ethics Statement

The studies involving human participants were reviewed and approved by Institutional Review Board of Yonsei University Health System (4–2019–0796). Written informed consent for participation was not required for this study in accordance with the national legislation and the institutional requirements.

## Author Contributions

Study conception and design: HKB, JSC; Provision of study materials or patients: JSC, MJ, WSK, KYC, BHO, MRR, KHK, C-KL, SJS; collection and assembly of data: HKB, JSC; data analysis and interpretation: HKB, JSC; manuscript writing: HKB, JSC; drafting or revising the article: all authors; All authors contributed to the article and approved the submitted version.

## Funding

This work was supported by a National Research Foundation of Korea (NRF) grant, funded by the Korea government (MSIT; grant no. 2019R1C1C1009359), as well as the Korea Medical Device Development Fund grant, funded by the Korea government (the Ministry of Science and ICT, the Ministry of Trade, Industry and Energy, the Ministry of Health & Welfare, the Ministry of Food and Drug Safety; grant no.: 202012E0102).

## Conflict of Interest

The authors declare that the research was conducted in the absence of any commercial or financial relationships that could be construed as a potential conflict of interest.
