# Supplementary material for: Prediction of Immune-Checkpoint Blockade Monotherapy Response in Patients With Melanoma Based on Easily Accessible Clinical Indicators
Source: Front Oncol. 2021 May 27;11:659754. doi: 10.3389/fonc.2021.659754 (PMC8190329; doi:10.3389/fonc.2021.659754)
Supplement: Supplementary file 1 [file Table_1.docx]

Supplementary Table 1. Treatment profiles of late responders

| Age/Sex | Subtype | irAE | PFS (m) | OS (m) | Last status | ICB | Subsequent treatment after ICB |
| --- | --- | --- | --- | --- | --- | --- | --- |
| 81/F | Uveal | Hypothyroidim | 2.7 | 4.3 | died of disease | Ipilimumab for 3 m | None |
| 62/F | Acral | Hypothyroidim | 34.1 | 34.1 | alive | Pembrolizumab for 24 m | None |
| 56/M | Acral | Hypothyroidim | 11.7 | 24.5 | alive | Pembrolizumab for 24 m | None |
| 68/M | Acral | Vitiligo | 2.0 | 23.5 | alive | Pembrolizumab for 3 m | #1. Darcarbazine for 17 m #2. Paclitaxel/Carboplatin for 3 m  10 courses of RT for recurrence sites |
| 65/F | Non-CSD | Vitiligo | 8.6 | 28.9 | alive | Pembrolizumab for 9 m | #1. Darcarbazine for 6 m #2. Paclitaxel/Carboplatin for 10 m  GKS and tumor removal for brain metastasis Axillary node dissection for nodal metastasis |
| 45/M | Non-CSD | Vitiligo | 1.8 | 68.3 | alive | Ipiliumab for 2 m | #1. Pembrolizumab for 24 m #2. Darcarbazine for 6 m #3. PanRAF inhibitor for 5 m #4. Dabrafenib + trametinib for 4 m #5. Nivolumab for 5 m   9 courses of RT and 5 courses of GKS |
| 55/F | Uveal | Vitiligo | 14.5 | 43.6 | died of disease | Nivolumab for 14 m | #1. Paclitaxel/Carboplatin for 9 m #2. Pembrolizumab for 4 m |

Abbreviations: CSD, chronic sun damage; GKS, gamma knife surgery; ICB, Immune checkpoint blocker; irAE, immune-related adverse event; m, month; OS; overall survival; PFS, progression-free survival; RT, radiotherapy

Supplementary Table 2. The number of patients according to the type of immune checkpoint blocker and the risk groups

|  | Group | | | |
| --- | --- | --- | --- | --- |
|  | A | B | C | D |
| Ipilimumab | 8 (18.6) | 12 (29.3) | 8 (22.2) | 2 (28.6) |
| Pembrolizumab | 34 (79.1) | 27 (65.9) | 25 (69.4) | 4 (57.1) |
| Nivolumab | 1 (2.3) | 2 (4.9) | 3 (8.3) | 1 (14.3) |
| Total | 43 | 41 | 36 | 7 |

Group A: favorable group (patients with normal baseline LDH level and no CNS/visceral metastasis); group B: unfavorable group (patients with elevated baseline LDH level or CNS/visceral metastasis) without lymphopenia within 3 months; group C: unfavorable group with lymphopenia within 3 months; group D: late responder (unfavorable group with immune-related hypothyroidism or vitiligo within 6 months)

The data represent the number (%).
